# Laparoscopic bariatric surgery training program model: gastric bypass

**DOI:** 10.1186/1471-2482-14-101

**Published:** 2014-12-03

**Authors:** Fabio R Thuler, Wilson R de Freitas, Elias J Ilias, Paulo Kassab, Carlos Alberto Malheiros

**Affiliations:** Department of Surgery of the Santa Casa Medical School, São Paulo, SP Brazil

**Keywords:** Bariatric surgery, Morbid obesity/surgery, Gastric bypass/education, Learning curve, Training, Laparoscopy, Inservice training, Staff development

## Abstract

**Background:**

Laparoscopy for bariatric surgery became the surgery of choice for surgeons worldwide. However, it is also more difficult to learn and has a great potential for complications. The specific training is fundamental to maintain the benefits without increasing the complications. This study presents a laparoscopic surgery training method for the treatment of obesity and to analyze its efficiency.

**Method:**

A training program for 36 surgeons with experience in open bariatric surgery was proposed, and the surgical results of their first laparoscopic surgeries were accompanied as for greater complications, such as death, intestinal obstruction, bleeding and fistula within the first 30 days.

**Results:**

Of the 36 surgeons who completed the program, thirteen who performed 403 surgeries were accompanied for 18 months to evaluate morbidity and mortality. There were 4 cases of greater complications (1%).

**Conclusions:**

The proposed program was efficient for this specific group of surgeons, as it permitted the participants to learn the procedure without increasing the initial complications in the learning curve.

## Background

Obesity in nowadays an endemic disease in the world and, consequently, an increase in the number of bariatric procedures is well-documented subjects in the literature [1, 2]. In January, 2012, the Brazilian National Health Agency included laparoscopic access for bariatric surgery in the list of procedures paid by the health insurance companies, which naturally induced an increase in the demand for this procedure and for training on the part of Brazilian surgeons. Because of that, any surgeons used to perform this procedure through laparotomy found themselves obligated to initiate the laparoscopic access training. Laparoscopy for bariatric surgery became the surgery of choice for surgeons worldwide, as it presents advantages in terms of surgical trauma, pain and early discharge from the hospital [2, 3]. It is also more difficult to learn and has a great potential for complications. The importance of specific training is fundamental to maintain the benefits without increasing the complications [4]. In early 2012, we initiated a training program in laparoscopic bariatric surgery for surgeons who already performed bariatric surgery through open access, with the intention of broadening the access and decreasing the number of complications related to the learning process for laparoscopy. The objective is to present a method of training in laparoscopic surgery for the treatment of obesity and to analyze its efficiency.

## Methods

### Surgeons (Trainees)

The present study was in compliance with the Declaration of Helsinki, and was approved by the ethics committee of the Santa Casa Medical School number 894.464. Written informed consent was obtained from the participants for the publication of this report and any accompanying images. The surgeons for the program were selected according to two items of criteria: those performing at least 3 bariatric surgeries per month and those having previous training in basic laparoscopic surgery (cholecystectomy). These surgeons registered and were sponsored through Johnson & Johnson Medical Brazil’s sales professionals in their cities. They were divided into 6 courses, each having 6 students, totalizing 36 surgeons from 6 different states and 34 cities. The course duration was two days.

### The program

The program was divided into three distinct stages, according to the Fit & Posner skills acquisition theory [5]: cognitive, integration and automation. In the cognitive phase, the procedure was divided into very distinct stages, explained and demonstrated by means of videos, which were edited step by step. In the integration phase, they received training in black boxes with pig stomachs, where they performed part of the procedure with training in sutures and stapling. Following that, the surgeons participated in three procedures performed by the program instructor, having then the opportunity to help and assist, reinforcing the standardization and the intraoperative details. In the last stage of automation, the surgeons scheduled an average of four surgeries (gastric bypass) in their home cities, with the participation of the instructor, until they could perform the complete procedure.

In order to make this possible, the two first stages were completed in two days:

On the first day, at the training center, the cognitive and simulated training phase was performed, which consisted of four hours of theoretical classes on laparoscopic themes: how to initiate bariatric surgery, technical standardization of the gastric bypass, standardization of the suturing technique, training with laparoscopic staplers and ultrasonic energy devices and sessions of complications in bariatric surgery.

The classes were interspersed with three morning hours of training in training boxes, which simulated the abdomen, and three more hours in the afternoon. Pig stomachs were utilized to do the training in sutures and anastomosis.

On the second day, three surgical procedures were performed on human beings with simultaneous transmission, highlighting each step in the technical systematization and the surgeons had the opportunity to participate in the surgical field and to get a feeling for the intraoperative difficulties. Each student entered one procedure and watched the other two in real time.

Subsequent to the training, the surgeons scheduled their surgeries in their cities of origin and were accompanied in their first procedures by an experienced surgeon from the training program until they were able to perform them with their own teams.

All of the participants received a descriptive booklet, a DVD with the technical standardization and non edited videos of the surgeries in order to review before they performed their tutored procedures.

### Technical standardization of suturing in the simulator box

The first step was the training to perform the internal sliding knot. After that, the execution and training for the closing of the two types of gaps in the stapling lines in the pig stomach was initiated: the transversal gap (simulating gastroenteroanastomosis,) and the longitudinal gap (simulating enteroanastomosis) with continuous suturing over a Fouchet tube for the gastroenteroanastomosis and with suturing with parallel hands for the enteroanastomosis.

### Evaluation of the training stage in the laboratory

At the end of the suture and anastomosis training program, the surgeons had performed a closing of the longitudinal and transversal gaps and the preceptor had applied an evaluation scale for surgical skills proposed by Martin J.A. OSATS [6]. In this evaluation, the score varied from 5 to 20, the minimum passing score being 12 to be approved for advancing to the next stage of the program. The students who did not achieve the minimum score redid the training until they reached the required score before advancing to training in the surgical field.

### Standardization of the surgical technique

The technical standardization of the chosen bypass has been the one utilized by the team since 2006 and was divided into seven stages to enhance the learning process. Each stage was discussed in classes and during real time surgeries. At the end of two days, the surgeons were familiar with the surgical procedure stages, with the materials they would use and with the surgical skills needed for the surgery to be performed. At this moment, the surgeons scheduled their surgeries in their home cities to be accompanied by the same instructor in two to eight surgeries until this instructor considered them as being able to perform the procedure using the OSAT scale [6]. The minimum score was considered to be 12 to initiate the procedure without tutorship.

#### Patient positioning

The patient was positioned with open arms and legs, with a anti-Trendelenburg of 30°.

#### Surgical team positioning

The French method was used, with the surgeon between the patient’s legs for the first part of the surgery: confection of a gastric pouch and gastroenteroanastomosis and changing positions for the confection of enteroanastomosis and closing of gaps.

#### Trocars insertion

The first incision was made 15 cm below the xiphoid, a little lateral to the median line to its left. Six trocars were introduced, 3 being five-millimeters, 1, ten-millimeter and 2, twelve-millimeters.

#### Making of the gastric pouch

A space was dissected between the first and second gastric vessels in the esser curvature, until accessing the retrogastric space 4 cm from the gastro oesophageal junction, utilizing an harmonic scalpel. The introduction of the stapler was made through the 12-mm trocar left of the surgeon through laparoscopic vision with a 45-mm blue cartridge using its whole extension. The distance between the Hiss angle and the end of the stapler is approximately 4 cm, measured with a grasper that allows to measure adequately.

To expose the Hiss angle, an articulated grasper was passed behind the stomach, exiting in the angle. After that we initiated the longitudinal stapling with 45-mm blue cartridge, 2 or 3 shots being necessary to reach the separation of the gastric pouch, which should have at least 1 cm of distance laterally from the esophagus.

Positioning of the bilio-pancreatic loop.

After identifying the Treitz angle, 70 cm of the jejunal loop was measured with a centimetered grasper and the terminal-lateral gastrojejunal anastomosis is performed.

#### Gastroenteroanastomosis

The gastroenteroanastomosis was performed in the posterior wall of the gastric pouch with a 45 mm blue cartridge stapler, leaving an anastomosis of 2 cm in diameter and a gap of 2.5 cm. The closing of the gastroenteroanastomosis was finished with a suture in a single layer PDS 3–0 thread, molded over a 36-French sizing bougie. After finalizing the anastomosis, we stapled the bilio-pancreatic for the confection of the Roux-en-Y.

#### Measurement of the alimentary loop and enteroanastomosis

At this time, the surgeon began operating from the right side of the patient with two lateral trocars, positioned in front of the operating field. The measurement of the loops was also made with the grasper. The latero-lateral stapled enteroanastomosis was performed 1 meter from the gastric anastomosis and also finished with a continuous suture in a single layer in the extramucosal plane with PDS 3.0.

#### Closing of the mesenteric gap

Only the mesenteric gap of the enteroanastomosis was closed with continuous 2–0 cotton suturing.

#### Surgical review

Every surgery went through a final review of stapling lines, sutures and loop positioning, correcting possible abnormalities and, if necessary, draining the cavity at this moment. The trocars were removed under laparoscopic vision, mainly the 12-mm ones.

### Selection criteria

For the performance of the procedures in their cities, selection criteria were adopted so as to facilitate the initial training:

- BMI under 45
- Anesthetic risk according to the American Society of Anesthesiology (ASA) II [7]
- Age under 60 years
- Women

## Results

In total, 6 courses with 6 participants each were taught, from March, 2012 to November, 2012, in alternated months. Some surgeons did not complete the training, as they did not have the surgical volume, which was consistent with the program or minimum infrastructure in their cities, such as videolaparoscopic equipment and adequate materials for surgical safety.

Of the 36 participants, we recovered the data on 13 one year after training by direct contact and the filling out of the protocol below. Only major complications, such as death, bleeding, intestinal obstruction and fistula in the first 30 days were considered. The 13 surgeons performed a total of 403 surgeries and presented with 4 cases of major complications, representing 1% of the complications, those being one fistula of clinical treatment, one enterorrhagia of clinical treatment which needed transfusion of 3 units of Red blood concentrate (RBC), one obstruction in the orifice of the trocar and one non-therapeutic laparotomy due to abdominal pain on the second postoperative day (Table 1).

**Table 1:** **Results of the training program**

Surgeon	Number of surgeries	Major complications (first 30 days)	Training scores (OSATS Scale)
A	32	1 conversion	14
B	19	0	12
C	30	0	15
D	35	0	10/12
E	25	0	14
F	27	0	16
G	6	0	16
H	56	0	16
I	60	1 fistula	12
J	40	1 obstruction	12
K	10	0	10/12
L	22	0	12
M	41	1 enterorrhagia	9/12

## Discussion

The proposed program was based on the experience acquired in preceptorships performed over 4 years in several Brazilian cities and states, in which it was possible to observe the technical difficulties of the surgeons. In Brazil, nowadays, we have to train surgeons that already perform open surgery, to perform the laparoscopic access. The program was designed for this reason and is not applicable for beginning surgeons or residents. The learning system with tutorships *in loco* for the teams to be trained is more difficult to perform and needs various procedures until the surgeon has obtained the necessary knowledge of standardization through repetition. According to the Fit & Posner theory of motor skill acquisition, we must go through the cognitive, integration and then the automation phases [5]. Several studies demonstrated the efficiency of laboratory training before beginning the procedure in an operating room, as well as providing lower cost training and better performances on the part of the participants, in comparison to those who did not undergo training [4, 8–10]. The training made it possible to substitute the cognitive and integration phases and anticipate the automation phase in models, which were similar to the procedure to be performed. The use of virtual reality did not show itself to be more efficient than the training in simulator boxes as for the acquisition of these skills. This must be considered, because the cost of training in virtual reality is higher [11, 12].

Several authors have shown that the training of surgical skills in simulators would anticipate learning and bring more confidence to the procedures. Iordens *et al.* showed that after appropriate training of residents in surgical abilities in laboratory and appropriate tutorial in initial cases the residents in his service showed results similar to those of the assistants, differing only as to the surgical time [8]. Zevin, performed a review on retrospective and prospective studies on the learning curve and drew attention to the greater incidence of complications before this training, leading us, once again, to consider the importance of this step [12].

Specifically about the bariatric surgery, a systematic review comparing the learning curve between surgeons that had undergone specific training in bariatric surgery and surgeons that had not, provided an already expected reduction in the morbidity and mortality in the group with previous training [4].

For this reason, we initiated the program with training in simulator boxes with a pig stomach for training in endosutures and stapling, interspersed with technical explanations on the procedure, materials and equipment.

The suturing training program aims to develop the suturing technique applied to the difficulties in the laparoscopic bariatric surgery positioning. Even a surgeon used to stitching in laparoscopy may have difficulty in performing the closing of the gaps created by the stapling, due to its peculiarities. Therefore, the objective is to simplify the suturing and systematize the technique to make the learning easier.

The proposed training was able to abbreviate the transfer of knowledge in the cognitive phase and integration in the laboratory for surgical skills training and made possible more suitable logistics, decreasing the number of trips necessary to train a team.

The learning curve, so well referenced in the literature in innumerable articles as being the number of procedures the surgeon needs to reach a plateau regarding time, conversion, complications and mortality, varies in bariatric surgery from 50 to 150 procedures [4, 8, 12]. Through this program, we were able to accompany 13 surgeons, in a total of 403 procedures in the initial phase of their experience In this period there is a greater risk of complications, as all are in their first 50 cases, below the so-called learning curve. Nevertheless they had a global complication rate similar to that related in the literature for surgeons above the learning curve [13–15].

The selection of the participants is an important step for the success of the program, as we understand that any surgeon may be trained for this surgery, but certainly needs a minimum of frequency to execute it, and that is why we believe that the number of procedures per month this surgeon performs, is more important than his or her knowledge of laparoscopy. Despite having trained 36 surgeons, four did not continue with their training, five were already members of other teams with experience in laparoscopic bariatric surgery, five did not request preceptorships and therefore were not accompanied, and for nine it was impossible to recover the data.

Another fundamental point of the program is the selection of the patient, as some papers show that men with BMIs over 50, have a higher complication rate [16]. Thus, there is no reason to initiate this procedure with the most difficult cases, as they not only multiply the chance of complications, but also demotivate the teams in training.

In spite of the importance of the surgical team, so well discussed at conferences, the proposed standardization perfectly allows the surgeon to perform the procedure with other surgeons having little experience in advanced laparoscopy. The reality of several cities in the Brazilian countryside does not permit the existence of teams exclusively dedicated to surgery for obesity.

The program itself has a pre-established model, but it must be adapted to the difficulties presented by the surgeons. Therefore, when we identify deficiencies in certain fundamentals, we allow for more time, so that that difficulty will be overcome. For example, in spite of endosuturing being the great obstacle for these surgeons, we know this skill can be well developed with oriented training and supervision. When we have a professional who presents with this difficulty, we train this person more in this fundamental. It is noteworthy that even though the fundamental of endosuturing can interfere greatly in the rate of complications, if the surgeon knows the standardization well, if the stapling is adequate, if the tissue manipulation is adequate, he or she can take more time to close the small orifice of the stapling, as the technical aspect, the safety and the results will be the same as for the surgeon who sutures rapidly.

## Conclusions

The proposed training program for laparoscopic bariatric surgery was efficient for this specific group of surgeons, as it permitted the participants to learn the procedure without increasing the number of initial complications in the so-called learning curve.

## Authors’ information

Fabio R. Thuler - Assistant Professor. Wilson R. Freitas Jr. - Assistant Professor. Elias J. Ilias - Assistant Professor. Paulo Kassab - Associate Professor. Carlos A. Malheiros – Full Professor and Chairman of Gastric Surgery Division.
